# Case report: Donor-derived CLL-1 chimeric antigen receptor T-cell therapy for relapsed/refractory acute myeloid leukemia bridging to allogeneic hematopoietic stem cell transplantation after remission

**DOI:** 10.3389/fimmu.2024.1389227

**Published:** 2024-05-13

**Authors:** Xiaojuan Miao, Yanrong Shuai, Ying Han, Nan Zhang, Yilan Liu, Hao Yao, Xiao Wang, Guangcui He, Dan Chen, Fangyi Fan, Alex H. Chang, Yi Su, Hai Yi

**Affiliations:** ^1^ Department of Hematology, People’s Liberation Army The General Hospital of Western Theater Command, Sichuan Clinical Research Center for Hematological Disease, Branch of National Clinical Research Center for Hematological Disease, Chengdu, Sichuan, China; ^2^ Shanghai YaKe Biotechnology Ltd., Shanghai, China; ^3^ Engineering Research Center of Gene Technology, Ministry of Education, Institute of Genetics, School of Life Sciences, Fudan University, Shanghai, China

**Keywords:** relapsed/refractory, acute myeloid leukemia, C-type lectin-like molecule 1, donor-derived chimeric antigen receptor T cells, allogeneic hematopoietic stem cell transplantation

## Abstract

**Background:**

Explore the efficacy and safety of donor-derived CLL-1 chimeric antigen receptor T-cell therapy (CAR-T) for relapsed/refractory acute myeloid leukemia (R/R AML) bridging to allogeneic hematopoietic stem cell transplantation (allo-HSCT) after remission.

**Case presentation:**

An adult R/R AML patient received an infusion of donor-derived CLL-1 CAR-T cells, and the conditioning regimen bridging to allo-HSCT was started immediately after remission on day 11 after CAR-T therapy upon transplantation. Then, routine post-HSCT monitoring of blood counts, bone marrow (BM) morphology, flow cytometry, graft-versus-host disease (GVHD) manifestations, and chimerism status were performed.

**Result:**

After CAR-T therapy, cytokine release syndrome was grade 1. On day 11 after CAR-T therapy, the BM morphology reached complete remission (CR), and the conditioning regimen bridging to allo-HSCT started. Leukocyte engraftment, complete donor chimerism, and platelet engraftment were observed on days +18, +23, and +26 post-allo-HSCT, respectively. The BM morphology showed CR and flow cytometry turned negative on day +23. The patient is currently at 4 months post-allo-HSCT with BM morphology CR, negative flow cytometry, complete donor chimerism, and no extramedullary relapse/GVHD.

**Conclusion:**

Donor-derived CLL-1 CAR-T is an effective and safe therapy for R/R AML, and immediate bridging to allo-HSCT after remission may better improve the long-term prognosis of R/R AML.

## 1 Introduction

Relapsed/refractory acute myeloid leukemia (R/R AML) has a low remission rate with chemotherapy and a high probability of relapse after salvage HSCT has been performed in the absence of remission (1–3). Therefore, it is challenging to regain remission before HSCT to achieve good conditions for successful hematopoietic stem cell transplantation (HSCT) and reduce the risk of subsequent relapse. In recent years, the success of CD19 chimeric antigen receptor T-cell (CAR-T) therapy in B-cell malignancies has led to the exploration of the feasibility of using CAR-T for the treatment of acute myeloid leukemia (AML) (4, 5).

C-type lectin-like molecule 1 (CLL-1) is a membrane protein that plays a pivotal role in the fight against infection and maintains homeostasis and self-tolerance by recognizing damage- and pathogen-associated molecular patterns that lead to the regulation of innate and adaptive immunity (6). Non-hematological tissues in humans express very low levels of CLL-1 (7). In the hematopoietic tree, CLL-1 is mainly expressed by almost all granulocytes and monocytes and by approximately 61.8% of their precursors, 41.6% of progenitors, and only 2.5% of CD34^+^CD38^−^ HSCs, but it is not expressed by T, B, and NK cells or erythrocytes or by their precursors (8). CLL-1 is also expressed by basophils, eosinophils, granulocytes, macrophages, and myeloid DCs (9). CLL-1 is also expressed in leukemic stem cells (LSCs), which have the ability to self-renew indefinitely and produce many daughter blast cells, representing one of the most important causes of leukemia relapse (10, 11). As a result, CLL-1 can serve as a marker of LSC and disease relapse. More importantly, CLL-1 is expressed by >80% of AML cells but not by normal HSC (12, 13), allowing CLL-1 to be considered an ideal druggable target for the treatment of AML.

A phase I/II clinical trial of autologous CLL-1 CAR-T therapy by Zhang et al. enrolled eight children with R/R AML, all of whom received autologous CLL-1 CAR-T therapy after a conditioning regimen with fludarabine and cyclophosphamide (Flu/Cy) (14). After Flu/Cy treatment, the patients experienced grade 1–2 cytokine release syndrome (CRS) with no fatal adverse events. Of these four children who achieved bone marrow morphology complete remission (CR) and minimal residual disease (MRD)-negative status, one child showed positive BM morphology and MRD, one child achieved CR with incomplete count recovery (CRi) with positive MRD, one child achieved partial remission (PR), and one child maintained stable disease (SD) status (14). In another phase I clinical study, Jin et al. enrolled 10 adult patients with R/R AML who received 1–2 × 10^6^/kg autologous CLL-1 CAR-T cells after Flu/Cy (15). All 10 patients developed CRS (low-grade in four patients and high-grade in six patients), none of these patients developed CAR-T therapy-related encephalopathy syndrome (CRES), and 70% of these patients achieved CR/CRi. All patients presented severe pancytopenia, which was attributed to the fact that CLL-1 was also expressed in normal granulocytes. Two patients died from severe infections caused by prolonged granulocyte deficiency (15). Therefore, bridging to HSCT was considered to rescue the resulting prolonged granulocyte deficiency.

As evidenced by current studies on immunotherapy with autologous CLL-1 CAR-T cells, CLL-1 is an effective target for the treatment of R/R AML, and bridging to HSCT is required after remission to rescue the subsequent granulocyte deficiency, reduce the risk of post-HSCT relapse, and improve long-term prognosis. However, some patients with R/R AML have extremely low autologous lymphocyte counts due to a high tumor load and are unable to provide autologous lymphocytes for the preparation of CAR-T cells. Li Z et al. analyzed 12 patients with R/R T-ALL/LBL (16). These patients obtained CR or PR through donor-derived CD7-CAR-T therapy bridging to allo-HSCT, and the OS and DFS at 6 months were 91.67% and 83.33%, respectively, and the allo-HSCT-related mortality (TRM) was 8.33%. This result showed the strong anti-leukemic effect and safety of donor-derived CD7-CAR-T combined with allo-HSCT (16). Therefore, we describe an adult patient with R/R AML to explore the efficacy and safety of donor-derived CLL-1 CAR-T therapy bridging to allo-HSCT from the same donor after remission.

## 2 Case description

### 2.1 Patient characteristics before CAR-T therapy

An 18-year-old male patient was admitted to a local hospital in June 2021 with a fever. After routine blood tests, bone marrow puncture, and other related examinations, this patient was diagnosed with AML with CEBPA double mutation and normal karyotype (classified as low risk according to the ELN 2022 risk stratification). Subsequently, the patient received four courses of chemotherapy (first course: 170 mg of cytarabine d1–d7 and 130 mg of daunorubicin d4–d6; second course: 500 mg of cytarabine q12h d1–d3; third and fourth course: 5,000 mg of cytarabine d1–d3). Bone marrow (BM) morphology showed CR and flow cytometry showed positive MRD at the end of the first course of chemotherapy. The MRD was detected based on this phenotype of CD34^+^CD117^+^HLA-DR^+^CD13^dim+^CD33^dim+^CD38^+^CD123^dim+^CD200^+^CLL-1^+^CD56^+^CD7^+^CD19^−^. The patient achieved CR and negative MRD since the second course of chemotherapy. During this period, a lumbar puncture was performed and no abnormalities were observed on routine cerebrospinal fluid, biochemistry, and flow cytometry examinations. The patient underwent autologous HSCT (auto-HSCT) on 6 January 2022. The conditioning regimen was busulfan/Flu/Cy/chidamide (150 mg of busulfan d1–d4, 50 mg of Flu d1–d5, 1,500 mg of cytarabine on d1–d5, and 30 mg of chidamide d1, d4, d8, d11). On 24 May 2022, routine blood tests revealed a white blood cell count of 1.14 × 10^9^/L, a hemoglobin concentration of 62 g/L, a platelet count of 3 × 10^9^/L, and 4% immature cells. BM morphology revealed 71% myeloid blasts, and flow cytometry showed abnormal myeloid primitive cells comprising approximately 77.74% of nucleated cells, with a phenotype of CD34^+^CD117^+^HLA-DR^+^CD13^dim+^CD33^dim+^CD38^+^CD123^dim+^CD200^+^CLL-1^+^CD6^+^CD7^+^CD19^−^. Chromosome karyotype analysis showed 45,XY,-9. Analysis of myeloid gene mutations identified a CEBPA double mutation. In summary, this patient was diagnosed with R/R AML with CEBPA double mutation and was classified as high risk according to the ELN 2022 risk stratification. Hence, the patient received one course of venetoclax and azacytidine chemotherapy (100 mg of venetoclax d1–d28 and 100 mg of azacytidine d1–d9) on 29 May 2022. BM morphology showed 61% primitive granulocytes on 4 July 2022. Flow cytometry showed 63.18% abnormal myeloid primitive cells suggesting a refractory disease.

### 2.2 CLL-1 CAR-T therapy bridging to allo-HSCT after remission

**Figure 1:**
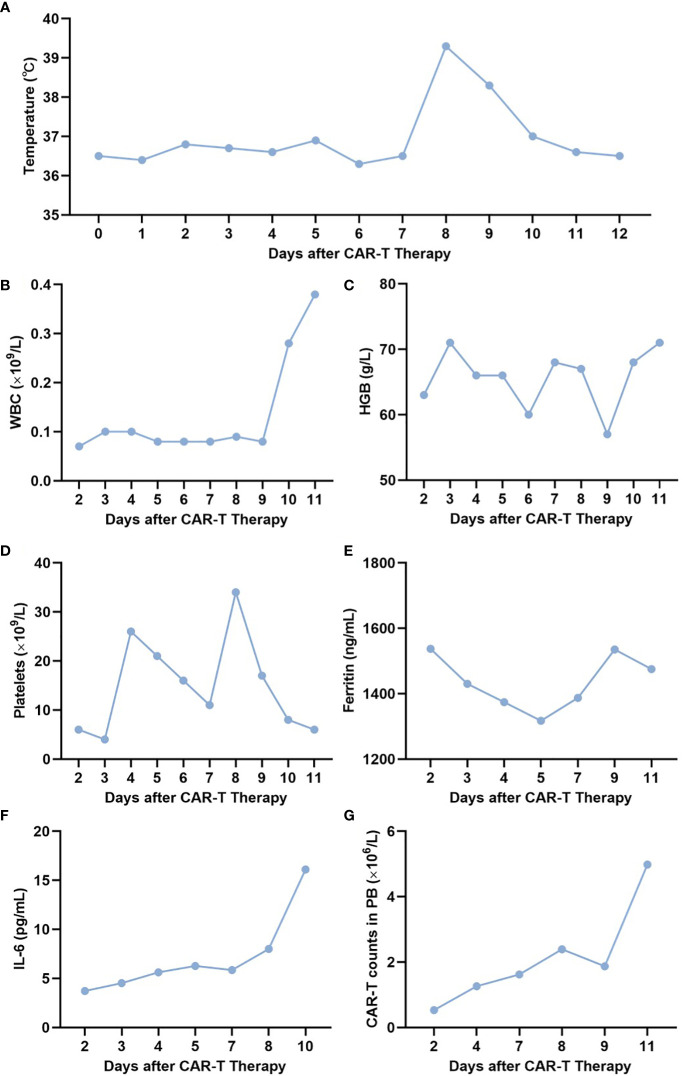
Related indexes of the patient at different time points. **(A–G)** Temperature **(A)**, WBC **(B)**, HB **(C)**, PLT **(D)**, ferritin **(E)**, IL-6 **(F)**, and CAR-T cell count **(G)** in the peripheral blood of the patient on days 2, 4, 6, 8, 10, and 12 after CAR-T therapy. WBC, white blood cell; HB, hemoglobin; PLT, platelet.

Given the refractoriness of the patient, we proposed that the patient should undergo donor-derived CLL-1 CAR-T therapy, and we obtained the patient’s understanding and consent. The donor was his 21-year-old older sister. We used the COM.TEC blood component separator (Fresenius, Bad Homburg, Germany) to collect the donor’s peripheral blood T cells, which subsequently were stimulated with magnetic beads coated with anti-CD3/CD28 antibodies (Thermo Fisher Scientific Massachusetts, United States of America) overnight. The patient received Flu/Cy therapy (50 mg of Flu for 3 days, 500 mg of Cy for 3 days) starting on 22 July 2022. Then, a total of 0.5 × 10^6^/kg of donor-derived CLL-1 CAR-T cells were infused on 1 August 2022 and on 4 August 2022, respectively. The CAR-T manufacturing protocol was performed as follows (17). Briefly, the CLL-1 CAR lentivirus was manufactured at our center under good manufacturing practice (GMP) standards. CLL-1 CAR-T cells were manufactured with donor-derived T cells transduced with CLL-1 CAR lentivirus. Transduction efficiency, defined as the percentage of CAR^+^ cells among CD3^+^ cells, and cell viability were determined just before infusion by flow cytometry and Trypan blue exclusion. Subsequently, vital signs, blood counts, cytokine levels, ferritin levels, peripheral blood CAR-T cell count, BM morphology, and flow cytometry were closely monitored. On day 8 after CAR-T therapy, the patient developed a fever with a maximum temperature of 39.2°C, which subsided on day 10 after 10 mg of intravenous dexamethasone (**Figure 1A**). No other adverse effects were observed, including blood pressure drop, capillary leak syndrome, CRES, gastrointestinal events, cardiovascular events, change in general conditions (fatigue, flu-like symptom, rash, and peripheral edema), laboratory values (AST increase, ALT increase, bilirubin increase, creatine increase, and LDH increase), or infection. CRS was grade 1. The patient had a severe reduction in whole blood cells (**Figures 1B–D**) with a mild increase in ferritin and interleukin-6 (IL-6) levels (**Figures 1E, F**). CAR-T cell counts in the peripheral blood are shown in **Figure 1G**. BM morphology on 12 August 2022 (day 11 after CAR-T therapy) showed an extreme reduction in BM proliferation with 5% primitive granulocytes. Flow cytometry revealed 0.29% abnormal myeloid primitive cells in the BM. A conditioning regimen including decitabine/cladribine/cytarabine/busulfan/semustine/ATG (300 μg of G-CSF d1–d5, 30 mg of decitabine d1–d5, 8.4 mg of cladribine d1–d5, 1,600 mg of cytarabine d1–d5, 180 mg of busulfan d1–d3, 400 mg of semustine d5, 200 mg of ATG d3, and 300 mg of ATG d4–d6) was carried out on 12 August 2022 (day 11 after CAR-T therapy), and the patient received donor stem cells [mononuclear cells (MNCs) 8 × 10^8^/kg, CD34 6.6 × 10^6^/kg, total nucleated cells (TNCs) 12.3 × 10^8^/kg, CD3 1.3 × 10^8^/kg] on 20 August 2022 (day 0 after allo-HSCT), with routine treatment of cyclosporine, mycophenolate mofetil, and short-course methotrexate (MTX) for graft-versus-host disease (GVHD) prevention. The donor was the same one described above, whose CMV status was negative and whose blood type was A+ (the patient’s blood type was AB+). The HLA compatibility between them was 8/12. Leukocyte engraftment, complete donor chimerism, and platelet engraftment were observed on days +18, +23, and +26 after allo-HSCT, respectively. BM showed CR and flow cytometry was negative on day +23 after allo-HSCT. No abnormalities were observed in routine cerebrospinal fluid, biochemistry, or flow cytometry examinations on day +32 after allo-HSCT. The patient is currently at 4 months post-allo-HSCT with bone marrow morphology CR, negative flow cytometry, complete donor chimerism, and no evidence of extramedullary relapse or GVHD.

## 3 Timeline

**Figure 2:**
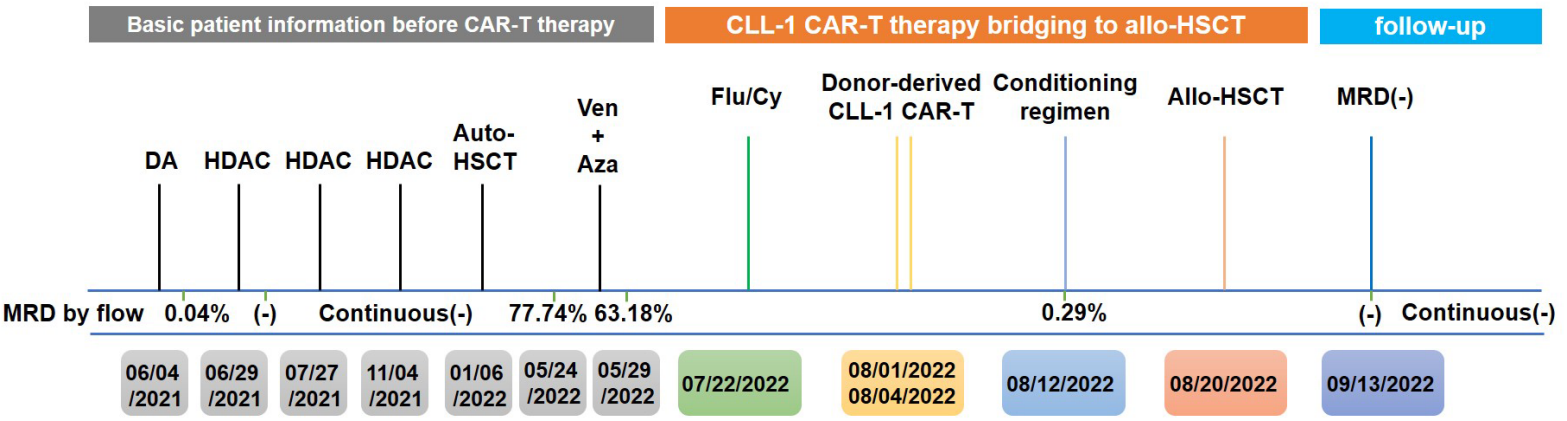
Timeline of the patient’s treatment and bone marrow assessment. DA, daunorubicin and cytarabine; HDAC, high-dose cytarabine; Ven, venetoclax; Aza, azacytidine; Flu, fludarabine; Cy, cyclophosphamide.

The timeline of the disease and treatment course of this patient is shown in **Figure 2**.

## 4 Discussion

AML is a highly heterogeneous group of malignant hematologic diseases. Although some low-risk patients achieve prolonged survival with chemotherapy, some patients do not benefit from chemotherapy and may progress to relapsed/refractory (R/R) AML. Treatment after relapse remains a challenge, especially for AML that relapses after HSCT, with no standard therapies and only a series of palliative treatments (18, 19). In recent years, the success of CD19 CAR-T therapy in B-cell malignancies has led to the exploration of the efficacy and safety of CAR-T therapy in AML, the targets of which include LewisY, CD44V6, CD33, CD123, and CLL-1 (4, 8, 20–24). Although more expressed in leukemic stem cells (LSCs), CD33 and CD123 are also frequently expressed in normal HSCs, and their suppression can lead to long-term or even permanent myelosuppression (25). The fact that CLL-1 is highly expressed in AML cells but is deficient in normal HSCs makes it an attractive target in CAR-T therapy for AML (26). The efficacy and safety of autologous CLL-1 CAR-T therapy have been demonstrated in previous clinical studies (14, 15). However, to date, no cases have described allogeneic donor-derived CLL-1 CAR-T therapy in R/R AML.

The patient we describe herein was initially diagnosed with CEBPA double-mutated AML (low risk). After achieving remission from induction chemotherapy, the patient underwent three courses of consolidation chemotherapy with sequential auto-HSCT. Relapse occurred within 6 months after auto-HSCT and then the patient was reclassified as R/R AML with CEBPA double mutation (high risk). Salvage chemotherapy with standard doses of venetoclax and azacytidine was performed after relapse; however, BM evaluation after chemotherapy indicated treatment failure. At this point, the patient was faced with the options of 1) immediate salvage allo-HSCT or 2) participation in a clinical trial of CAR-T therapy. However, the disease status at the time of allo-HSCT was closely related to the outcome after HSCT. Allo-HSCT is most effective in patients who reached CR with the lowest relapse rate since there is sufficient time to establish a strong graft-versus-leukemia (GVL) effect in such cases (2, 3). Therefore, immediate salvage allo-HSCT was not the optimal choice for this patient. The BM immunophenotyping of this patient revealed high CLL-1 expression by tumor cells on 24 May 2022. Therefore, participation in the CLL-1 CAR-T clinical trial was a feasible option. However, this patient was in a state of AML relapse and had an extremely low peripheral blood lymphocyte count, which made it difficult to collect sufficient autologous lymphocytes for the preparation of CAR-T cells. The results of Zhi Hui Li et al. showed strong anti-leukemia effect and safety of donor-derived CAR-T combined with allogenic HSCT (16). For this patient, we considered administering donor-derived CLL-1 CAR-T therapy bridging to allo-HSCT immediately after remission. After a conditioning regimen with Flu/Cy and infusion of donor-derived CLL-1 CAR-T cells, this patient developed grade 1 CRS on day 8 after CAR-T therapy. On day 11 after CAR-T therapy, BM morphology showed CR and flow cytometry showed 0.29% abnormal myeloid primitive cells. This case fully demonstrated that donor-derived CLL-1 CAR-T therapy has significant efficacy and good safety advantages, which can create favorable conditions for a transition to allo-HSCT. A routine conditioning regimen before allo-HSCT was started immediately after remission, followed by donor stem cell infusion and treatments to prevent GVHD. The patient recovered well after allo-HSCT and has a positive MRD status with no GVHD or extramedullary relapse manifestations at follow-up to date. These results demonstrated the safety and efficacy of donor-derived CLL-1 CAR-T therapy bridging to allo-HSCT immediately after remission.

The successful treatment of this patient indicates that donor-derived CLL-1 CAR-T therapy for R/R AML to achieve remission followed by immediate bridging to allo-HSCT is effective with mild and manageable adverse effects, thus providing new avenues for the treatment of R/R AML.

## Data availability statement

The original contributions presented in the study are included in the article/supplementary material. Further inquiries can be directed to the corresponding authors.

## Ethics statement

The studies involving humans were approved by the Ethics Committee of the People’s Liberation Army The General Hospital of Western Theater Command (Chengdu, China). The studies were conducted in accordance with the local legislation and institutional requirements. The participants provided their written informed consent to participate in this study. Written informed consent was obtained from the individual(s) for the publication of any potentially identifiable images or data included in this article. Written informed consent was obtained from the participant/patient(s) for the publication of this case report.

## Author contributions

XM: Investigation, Writing – original draft. YRS: Investigation, Writing – original draft. YH: Investigation, Writing – original draft. NZ: Investigation, Writing – review & editing. YL: Investigation, Writing – original draft. HYa: Investigation, Writing – original draft. XW: Investigation, Writing – original draft. GH: Investigation, Writing – original draft. DC: Investigation, Writing – original draft. FF: Investigation, Writing – original draft. AC: Investigation, Writing – review & editing. YS: Investigation, Writing – review & editing. HY: Funding acquisition, Investigation, Writing – review & editing.
